# Social Participation Considered as Meaningful in old age − the Perceptions of Senior Housing Residents in Finland

**DOI:** 10.1007/s12126-023-09522-z

**Published:** 2023-02-21

**Authors:** Ann-Louise Sirén, Marjaana Seppänen, Mikaela B. von Bonsdorff

**Affiliations:** 1 Folkhälsan Research Center, Social Gerontology, Helsinki, Finland; 2 Faculty of Social Sciences, University of Helsinki, Helsinki, Finland; 3 Gerontology Research Center and Department of Health Sciences, University of Jyväskylä, Jyväskylä, Finland

**Keywords:** Social participation, Meaningfulness, Senior housing, Autonomy, Quality of life

## Abstract

As populations across the world age, there is a recognised need for promoting social participation in older adults. Previous studies related to social participation have addressed that interactions perceived as meaningful may improve quality of life in old age. However, what is less clear is the nature of such participation from the perspective of older adults, as the vast majority of studies have been quantitative. The present study aimed to explore what characterises social participation that contributes to a meaningful everyday life, from the viewpoint of independently living Finnish older adults. Thematic analysis was used as an interpretative method drawing on semi-structured in-depth interviews with six residents aged 82 to 97 years from one senior housing facility. The analysis showed that social participation perceived as meaningful involved caring reciprocal interactions with people they connected with; having the freedom to make autonomous decisions and influence matters that affected their own or others’ everyday life; and, on a more abstract level, feeling significant as a person. It furthermore fostered independence and companionship as well as reduced loneliness. To describe social participation that is perceived as meaningful from a theoretical perspective, we used Levasseur and colleagues’ (2010) taxonomy and found that such involvement creates a sense of connectedness, a sense of belonging and relates to the concepts of social integration, social networking and social engagement. This type of involvement is associated with enhanced quality of life and a more meaningful life, highlighting the importance of creating environments where older adults can socially connect.

## Introduction

Today, we are living longer than ever before – a trend that necessitates societies to take action to promote social participation throughout the life course (World Health Organization [WHO], 2015). Although social participation is a term frequently used in the literature, a generally accepted definition of the concept is lacking (Aroogh & Shahboulaghi, 2020). This study utilises the concept of social participation as first proposed by Levasseur et al. (2010), who developed a taxonomy to differentiate between six levels of involvement in social activities. While participation encompasses all levels, only levels three through six are considered to be social participation because the two lowest levels only include distal involvement and are basic-needs oriented, such as doing activities alone or in parallel with other people (Levasseur et al., 2010). Level three is socially oriented and includes, for example, talking with friends or neighbours without doing a specific activity together; level four is task oriented and includes participating in activities together with others, such as hobby clubs or group activities organised in the community; level five includes helping others and volunteering; and level six includes society-oriented activities, such as being involved in political parties or organisations that allow individuals to contribute more broadly to society (Levasseur et al., 2010). Social participation in old age contributes to enhanced quality of life (Liu et al., 2020; Palmes et al., 2021; Roberts & Adams, 2018). Nevertheless, older adults are more likely to take part in social interaction that they perceive as meaningful (Mohler & Miller, 2020; Roberts & Adams, 2018); involvement in worthwhile social participation thus makes life more meaningful (Steptoe & Fancourt, 2019). To promote older adults’ social participation, it is therefore important to increase meaningful involvement. This paper seeks to explore what constitutes social participation that contributes to a meaningful everyday life, from the viewpoint of independently living Finnish older adults.

### Previous Research on Social Participation in old age

A key to promoting social participation is the identification of inhibiting and enabling factors (Cachadinha et al., 2011; De Coninck et al., 2021). A recent systematic review by Townsend et al. (2021) identified four main themes that related to decreased or increased social participation, namely demographic factors, individual/internal factors, environmental factors and social network sizes. The authors concluded that barriers can be mitigated or resolved through utilisation of facilitating factors; these enabling factors include pre-existing supportive networks as well as neighbourhood cohesion and accessibility. Mohler & Miller (2020) make a similar point in their qualitative study of facilitators and barriers to social participation among assisted living residents. They found that a major barrier for social participation was a lack of meaningful activities, as few of the offered activities were thought out. However, utilising strategies that match with the residents’ interests and functional and health needs can increase assisted living residents’ motivation to engage in enjoyable social activities as well as facilitate the development of meaningful relationships.

Another aspect of social participation that has been investigated in recent years is how different types of involvement influence ageing. Abe et al. (2020) found that older adults involved in neighbourhood associations had lower rates of frailty. The authors suggested that encouraging older adults to participate in the local community, such as participation in sports clubs or groups, can prevent early stages of frailty. In the same vein, a qualitative study by Duppen et al. (2020) described how casual social interaction with neighbours and others passing through the neighbourhood was important to frail older adults. This type of low-key social participation can generate feelings of belonging in the neighbourhood and enable participation in society, despite frailty. They suggested that neighbourhood participation may be more important for older adults than engaging in a specific social activity, as these social contacts promote well-being and social participation. One longitudinal study by Amagasa et al. (2017) investigated the relationships between different types of social participation and psychological distress in community-dwelling older adults. The results showed that more community involvement – volunteer activities, community events and clubs for older adults – was related to lower risk of psychological distress – but only in older women. Amagasa and colleagues suggested that community involvement contributes to improved mental health because it provides a sense of meaning in life and opportunities for social support perceived as satisfying. In a study by Ashida et al. (2016), data from a large cohort study were analysed to investigate whether the level of socio-economic status was associated with engagement in social participation, types of interaction and future need of long-term care. It was concluded that the types of social activities varied according to socio-economic status – older adults with high socio-economic status more often participated in sports groups and hobby groups and had a facilitating role compared to older adults with low socio-economic status. More involvement in such activities reduces the risk for functional disability and, thus, future need for long-term care.

What we know about social participation is largely based on quantitative studies. Most of these studies have typically ignored defining social participation. Defining the concept helps distinguish social participation from other related concepts (Aroogh & Shahboulaghi, 2020) and helps researchers to select appropriate instruments to measure social participation. Also, defining social participation makes it easier to compare results from different studies (Levasseur et al., 2010). While there is a role for quantitative approaches in the efforts to promote social participation, a disadvantage of many quantitative studies is the use of measurement scales that may not be in line with their definition of social participation or valid for certain populations. For example, Feng et al. (2020) pointed out that social participation may have been underestimated in samples including Chinese older adults because of a mismatch between the selected measurement scale and study sample. Choosing a measurement that focuses on formal social participation, such as sports clubs, is unsuitable because it is uncommon for older adults in China to join formal social organisations. This view is supported by Liu et al. (2020) who writes that the perceptions of what social participation comprises vary between different social and cultural contexts. To promote social participation in different populations, it is therefore important to complement quantitative methods with a qualitative approach, which can be more useful for identifying and characterising social participation as the study participants’ answers are not restricted by fixed-alternative questions.

### Senior Housing and Social Participation

Senior housing was considered to be a suitable setting for this study because this type of non-institutional housing is intended for older adults who can still live independently (Lahti et al., 2021) and with the aim to promote social participation (Carroll & Qualls, 2014; Jolanki, 2021; Roberts & Adams, 2018; Shippee, 2012). A growing number of Finnish older adults are relocating to senior housing facilities (Lotvonen et al., 2018), in Finland referred to as senior houses (Tyvimaa, 2011). This type of independent living is targeted for individuals who receive a pension, generally adults aged 55 years or older who manage to live independently – senior housing residents are able to make their own decisions and take care of themselves (Lotvonen et al., 2018). A difference between senior housing and assisted living or skilled nursing facilities is the level of care provided; senior housing residents generally have fewer health issues and require services that offer less support (Carroll & Qualls, 2014). Senior houses are built in safe neighbourhoods and are typically near public services and recreational areas (Tyvimaa & Kemp, 2011). The costs for living in a senior house vary, depending on location, services offered and whether it is a rental apartment or a condominium (Tyvimaa, 2010). Many senior housing residents are widowed, and women typically outnumber men (Lahti et al., 2021; Tyvimaa & Kemp, 2011).

Surprisingly, social participation has not yet been closely investigated in independent living contexts. While no studies have been found that aim to explore social participation in senior housing populations, several studies have shown that it plays an important role in senior housing residents’ lives. For example, the opportunities for social participation that senior housing communities provide may reduce feelings of loneliness (Jeste et al., 2019; Jolanki, 2021; Tyvimaa & Kemp, 2011). However, the loss of one’s spouse can cause a sense of loneliness despite opportunities for social interactions (Lotvonen et al., 2018); women who reside in senior houses are more often than men widowed, which can explain the higher percentage of women reporting loneliness (Lahti et al., 2021). To reduce loneliness, Fang et al. (2016) propose that available social activities must reflect the diverse needs and interests of senior housing residents to be appealing to engage in. Regular interaction with other residents makes it possible to develop friendships (Carroll & Qualls, 2014; Lotvonen et al., 2018). According to Roberts & Adams (2018), engaging in meaningful social activities makes it easier for senior housing residents to develop new important friendships and preserve a good quality of life. In a qualitative study investigating how a workshop method could help future senior housing residents plan their living environment, Henning et al. (2009) found that it is important to involve the residents in the development of their housing because it contributes to new social networks and a sense of meaningfulness. The benefits of involving senior housing residents in decision-making processes have been reported in several studies (e.g., Jenkins et al., 2002; Tyvimaa, 2011). Involving residents in the planning of social activities can motivate individuals who are less prone to participate because they find the activities meaningful (Roberts & Adams, 2018). Another important aspect of decision making for senior housing residents is having the choice to decide to what extent they engage in social activities with others; independent living allows residents to maintain their privacy as well as have opportunities to take part in social activities (Jolanki, 2021; Lotvonen et al., 2018).

This brief summary of the literature shows that while some researchers have addressed that certain types of involvement in social activities are meaningful, no studies have been found that attempt to investigate what actually characterises such participation in old age. The summary also highlights the need for complementing quantitative methods with qualitative approaches and adequately investigating social participation in independent living contexts such as senior housing communities. The current study aimed to address the identified gaps in the existing literature by exploring senior housing residents’ perceptions of social participation that contributes to a meaningful everyday life using a qualitative methodology.

## Methods

This study is part of a larger ongoing research project, the BoAktiv Study, which has been approved by the ethical review board in Humanities and Social and Behavioural Sciences, Helsinki University, Finland. Briefly, the larger study investigates health, function, social participation and sense of community and how these factors correlate with active ageing among Finnish senior housing residents. It also investigates how these factors correlate with health-related quality of life and symptoms of depression. Furthermore, information about health and well-being among Finnish senior housing residents is presented. Results from the larger study can be found in Lahti et al. (2021).

To gain deeper insight into social participation that is meaningful and how it is described by older adults, face-to-face semi-structured in-depth interviews were conducted with senior housing residents. Participants were recruited from one small-sized senior house (≤ 49 apartments) in southwest Finland operated by Folkhälsan, a non-profit organisation providing social and health services to Finnish citizens at all stages of life (Folkhälsan, n.d.). The senior house consisted of condominiums and rental apartments; approximately two-thirds of the apartments were condominiums. Both the lunch restaurant and activities organised at the senior house were open to the public. Although all residents were Finns, most of them had Swedish as their first language, which along with Finnish is an official language in Finland (Weaver, n.d.). The participants lived independently but could purchase services from the assisted living unit in the same building. Various activities such as aqua aerobics, gymnastics for older adults and mental gymnastics were organised by Folkhälsan’s activity staff. Many of the senior housing residents had pronounced decline in functional ability, were women and were widowed.

The study inclusion criteria comprised residents aged ≥ 65 years without diagnosed memory disorders. Permission to carry out the study was given by the senior housing manager. All residents in the senior house received an information letter about the study that included an invitation to participate. It was explained in the information letter that the aim of the study was to get a deeper understanding of senior housing residents’ perceptions on social participation and a meaningful life, two central aspects of quality of life. A meeting was then held in the common dining hall to provide more detailed information about the study. In total, eight residents attended the meeting and five signed an informed consent. Two of the attendees were under the age of 65 and did not meet our eligibility criteria, and one attendee felt she was too old and lacked the energy to participate. Residents who had not attended the meeting were informed that more participants were needed, and this resulted in one additional consent.

The participants were aged 82 to 97, and five of the six residents who agreed to participate were women. Three of the participants lived in condominiums and three in rental apartments. All participants were widowed and experienced various age-related conditions – the most common were hearing loss, impaired vision and limited mobility. Table 1 summarises the participants’ characteristics.

**Table 1:** Characteristics of the participants

	All, N = 6
***First language***	
Swedish	6
***Sex***	
Women	5
Men	1
***Age***	
80–85	1
85–90	4
95–100	1
***Years in school***	
9–10	2
11–12	2
13–14	1
15–16	1
***Health status***	
Diabetes type II	1
Forgetfulness/memory problems	1
Hearing loss	3
Low vision	5
Loss of tactile perception	1
Osteoarthritis	2
***Reduced ability to walk/inablity to walk***	
Walker	3
Wheelchair	1
***Assistance with activities of daily living***	
Showering	3
Getting in and out of bed	1

An interview protocol was composed and included background questions such as ‘How do you feel today physically/mentally?’ and ‘Do you have any chronic conditions?’ The interview also used open-ended questions such as ‘What does social participation mean to you personally?’ and ‘What do you personally find meaningful in everyday life?’ All participants were asked the questions formulated for the protocol. Based on their answer to a given question, different follow-up questions were asked to obtain more detailed information that was complemented by reflective listening. Two pilot interviews were performed with older adults who were familiar with senior housing. These pilot interviews were used to check whether the open-ended questions were comprehensible, if amendments were needed and to estimate the length of the interviews. All interviews were conducted in Swedish. The interviews were conducted by the first author because of her extensive interviewing experience from various public health projects. The interviews were carried out in the participants’ apartments, and the length of the interviews ranged from 69 to 138 min. All interviews were audio recorded and conducted between mid-March and mid-April 2019.

Thematic analysis, as described by Nowell et al. (2017), was chosen as the interpretive method for this study. This method was used to get a deeper understanding of the interviewees’ perceptions of social participation that is meaningful. The approach provided a systematic structure that both enabled access to the participants’ lived experiences and created a deeper level of abstraction during the process of theme development. The audio-recorded interviews were transcribed verbatim by the first author, who also conducted the three initial phases of the thematic analysis – that is, familiarising with the data, generating initial codes and searching for themes. Excel spreadsheets were used as a tool to manage data that were extracted from the transcripts during the theme development.

The transcripts were read iteratively to gain familiarity with the content and establish an overall picture of the data. Ideas about what was interesting about the data in each transcript were noted by inserting a comment in the margin of the Word document. Next, segments of text that were considered to relate to the participants’ perceptions of social participation and a meaningful everyday life were inserted into Excel. The analysis process was guided by an abductive approach that allowed us to explore less obvious findings that deviated from the original theoretical frame (Meyer & Lunnay, 2013). In the following phase, segments of text that related to the same theme were clustered together. Each set of texts with a shared theme was condensed and then labelled with a code that reflected the first author’s thoughts about the data. Similar codes were highlighted with the same colour. An iterative process generated new ideas about what was interesting to analyse.

Before proceeding to search for themes, the first author visited each study participant at their apartment and asked them whether the initial interpretations agreed with what they had intended to convey about social participation that added meaning to life. The aim with involving the participants at this stage of the analysis was to check whether the first author’s own interests and background had affected the choice of central codes. With a professional and personal interest in physical activity, the first author had a feeling that it had influenced the choice of labelling a code to physical activity; the participants’ feedback confirmed that although physical activity was important to preserve their health, it was less central in the context of social participation that is considered meaningful. Employing reflexivity was thus helpful to avoid projecting the researcher’s own preunderstanding and get a deeper understanding of the participants’ perceptions of meaningful social participation (Berger, 2015).

Once the potentially relevant codes had been developed, they were extracted into themes. In addition to identifying patterns connected to what the participants had explicitly expressed, the first author also searched for underlying meanings about what the participants wanted to communicate but had not been explicitly stated.

In the next phase of the analysis, it became clear that several themes were interconnected and related to one another but captured different characteristics of social participation that was perceived as meaningful. However, some themes had too little data to support them while other themes had to be separated to increase the richness of the data. Subsequently, the first author revisited the raw data to identify which themes should be regarded as findings that reflected the participants’ perceptions in relation to the research question. Altogether, four themes were identified. In the process, a significant overarching pattern in each transcript was also identified, summarising the themes on a more abstract level, including different dimensions of wanting to feel and be valued as an individual – to feel significant. These dimensions of feeling significant as a person formed an overarching theme, that was named *I matter*. At this stage of the analysis, the second author, an experienced qualitative researcher, independently reviewed the themes to determine whether the summaries of each theme were coherent, the labelling of the themes accurately reflected the content and refinements were needed. The two authors agreed that the names of the themes should be re-labelled, and repositioning some of the findings in each theme would make them more comprehensible. To confirm whether the overarching theme, I matter, reflected the participants’ voices, the first author once again visited each one of them at their apartment to ask them. Even though the participants said that they had not thought of it in those terms, they agreed with the interpretation of their narratives. All developed themes are summarised in Table 2.

**Table 2:** Overview of the data analysis process and developed themes

Examples of excerpts from raw data	Codes	Themes	Overarching theme
Our daughter has three children – two boys and one girl. The youngest boy… it has become a tradition that I call him every day at four-thirty in the afternoon. And he panics if grandmother doesn’t call him on time or answers the phone, but usually I try to make sure that I call him at four-thirty on the dot (participant 5). Well I don’t talk much myself but I listen to what others talk about and when they have memory lapses I help out (participant 6).	Caring Support Help out	Caring about and helping one another	
I definitely think that everyone should get together more and do something together, and I have thought so before too (participant 1). I prefer to socialise in small groups because it’s easier to build new connections. I have a neighbour I socialise with one-on-one, we have mutual friends from university that were all quite homogeneous, everyone was about the same age and on the same page (participant 6).	Connect Companionship To have things in common. Understand each other.	Connecting with others	
			I matter
That I get to decide for myself. Yes, I don’t want to be like a parcel that is put here and there. But you can’t... one thinks about the future, how one will manage when one gets frailer and so on ... no, but I want to be able to enjoy things that interest me or enjoy listening to music I like (participant 3). Then it’s meaningful, yes, that’s right. As it is now, I am fully independent, no one can come here and command me... no one gives me unwanted good advice (participant 6).	Freedom Choice	Free to decide for oneself	
I don’t think I can and have the energy to influence any longer, although I think everyone should interact more with one another (participant 1). We used to have quarterly meetings and the senior housing manager was there. I can go downstairs and talk to her too if there’s anything I want to discuss (participant 2).	Influence Opinions	Influence matters regarding the living environment	

## Findings

In this section, we present the developed themes – four themes and one overarching theme – that represent how the study participants perceived the characteristics of social participation that is meaningful. While the themes *caring about and helping one another*; *connecting with others*; *free to decide for oneself*; and *influencing matters regarding the living environment* represent explicit patterns that distinguish them from one another, the overarching theme *I matter* represents the underlying pattern that recurred in all themes − different dimensions of feeling significant as an individual. What these dimensions encompass will be further described when presenting the developed themes. Henceforth, our findings are presented with summaries and quotations.

### Caring About and Helping One Another

When the participants described what characterised social participation that was meaningful, they often mentioned social interaction that involved helping one another. This type of interaction, particularly with fellow residents, children and, to some extent, grandchildren, meant giving and receiving practical or emotional support and having a caring attitude towards each other. To exemplify, one participant stated that when she had severe depression and feelings of hopelessness, her husband, daughter and grandchildren remained by her side and helped her to get professional help. They expressed concern for her and were worried that they would lose the close bond they had before the depression had developed. Her friends at the senior house also remained by her side during her difficult time. Thus, because of the support the participant received from people who cared about her, in combination with medication, she eventually recovered and felt like her old self. The participant declared that now that she had recovered, she was helping fellow residents who were unable to fully manage on their own, particularly those who had physical limitations, and commented on this by saying:

Well, when I don’t have a walker… there was this one person who always came to visit and would just have a splash of coffee and that was my job [to get the coffee]. And now I have a friend who wants the whole mug full, and it is my job to bring her coffee; you see, she is in a wheelchair. It is good if you can just help out (participant 5).

This example shows that providing peer support could make everyday life easier or better for others, which in turn contributes to a more satisfying social life because they could continue to take part in social activities despite declining functional ability. One participant illustrated this by saying:

And that’s why we have a resident here who organises the dinner parties, and it’s once a month. And as a native here in town, she knows a lot of chefs and raises some money to hire them to cook… no, in the beginning several of us helped to organise, but in the end, she completely took over because she has her assistants who can help. In the past, everyone helped out, someone cooked, someone set the table. But these days we come to an already set table (participant 1).

Similarly, some participants stated that visits from their children provided opportunities to engage in activities they enjoyed. For example, one participant who had decided to stop driving because of old age often asked her daughter to take her for a drive. Others said that they would go out for dinner with their children when they came to visit. Overall, the participants felt that their children genuinely cared about them and helped them with duties or errands that they found difficult on their own, such as paying bills, going grocery shopping and buying clothes. Receiving practical support from their children also allowed greater independence. One of the participants, who used a wheelchair, said that her children had helped her modify her apartment so that she could access the balcony and use the kitchen countertop; this practical help from her children gave her the freedom to get fresh air independently and enjoy a cup of coffee from the coffee maker.

While the participants’ children mainly provided practical support to their parents, the participants described that it was more common for them to provide emotional support to their children. As one participant described it:

I should be an example to my children and be there for them. I think that my children should have confidence in me, that they should also tell me what has gone wrong or if something has happened to them that they would say just as it is, good or bad. Then I can help them (participant 4).

As indicated above, interactions with their children were based on reciprocity and respect. They understood that their children had busy lives but still made efforts to provide care for their ageing parents. Therefore, the participants felt that it was important to reciprocate with caring gestures. One participant said that she regularly treated her family to dinner at a restaurant in town. Another participant had begun to buy ready-made meals when her children came to visit because decreased functional ability restricted her from helping them cook dinner. Having respect for each other’s choices was also important. Several of our participants said that they never locked their front door at night, and, despite safety concerns, their children accepted their parent’s choice.

Regarding providing emotional support, the participants said that, in addition to being caring towards their children, it was important to show care for other senior housing residents. For example, one participant described that she had decided to socialise with a fellow resident who had expressed that he felt lonely after the death of his wife several years earlier. The interactions were constructive for both of them as their friendship was free of obligations or pressure.

The participants voiced a sincere concern for other senior housing residents’ well-being and wanted to help if they knew that someone needed their assistance. To give an example, if a fellow resident had not been seen in a while, regardless of if it was someone they knew well or an acquaintance, some of the participants said that they tried to find out whether this person was safe or needed help. If they knew the person well enough, they would check in on them. It was a way to look out for each other and made the participants feel safe.

### Connecting with Others

A recurring theme in our participants’ accounts of social participation perceived as meaningful was the importance of connecting, or bonding, with those they interacted with. They found it meaningful to interact with people they had something in common with, such as having shared experiences, mutual friends or similar interests or values that united them. Such an interaction contributed to a sense of belonging and was described as fitting in or being accepted for who they were, despite their imperfections – they could be themselves and felt safe enough to be honest, show vulnerability, talk about their own shortcomings or laugh when something unintentionally happened. Thus, it was important for the participants that the individuals they chose to spend time with had a non-judgmental attitude towards them.

However, the majority of the participants voiced that they longed to build new social connections because most of their friends they previously had close social ties to were dead. They expressed that the lack of companionship made everyday life less meaningful. One participant declared that:

Meaningfulness to me is probably relationships with friends and acquaintances … I think that’s important. But as I said, at this age when one after the other is dying… well, I’d say so. It’s just the relationships that are important. Where I used to live I socialised a lot, but then the contacts became less frequent of course over the years and… Well, so I have this one person but … well we don’t argue, and we have known each other and started school at the same time… but I can’t find that right connection with her (participant 1).

Another participant expressed a similar thought regarding the lack of meaningful social connections and also emphasised that it was a source of loneliness:

But it’s just at my age that I have two good friends, and both have died, that is natural, I’m 90 years old so most of my friends are gone. And I had some good friends, we could laugh when something did not go as planned, we planned and then it failed. So, we had the same sense of humor. I had some friends, and my husband and I understood each other. Now I don’t have anyone (participant 4).

These examples clearly show that social interaction only was meaningful for our participants if they felt that they socially connected, and a lack of companionship produced feelings of loneliness. To reduce involuntary loneliness, they attended activities that provided opportunities to get acquainted with new people they could connect with. Although it sometimes seemed to the participants that they had little in common with their fellow residents, most of them who had participated in social activities had found someone who shared their interests, such as culture or good food. These were people they spent much time with and felt a sense of companionship with.

### Free to Decide for Oneself

A subject that the interviewees highlighted and kept returning to was having the freedom to decide for themselves, which was enabled through independence and being in control of choosing their own social contexts and interactions with people, without direction from others. In other words, personal autonomy was highly valued. One aspect of autonomy related to physical and cognitive functions. The participants chose to take part in activities that were physically or mentally stimulating as a means to maintain independence. The main reasons they stated for staying active were to avoid becoming too dependent on other people or being forced to move from their current home to a long-term care facility. Nonetheless, most of the participants said that they were particularly grateful for still having their cognitive capacity intact, partly because it meant that they could take part in and follow conversations as well as help others who had memory problems but also because it allowed them to have their privacy. Although they enjoyed interacting with others, it was equally important that they had the authority to decide when they wanted privacy. On a related note, apart from choosing who to interact with, it was also important that they had the freedom to choose which activities to participate in. One participant exemplified this by saying:

Sometimes I feel that I should stay in instead of participating in an activity, since I had been at one the day before. But then I reconsider and decide that I want to go anyway. Everyone else will be there (participant 6).

Another aspect of autonomy concerned having the freedom to continue participating in enriching activities with people they cared about, such as eating out or attending cultural events. Having the ability to choose their own activities allowed the participants to stay social and spend time with people they enjoyed. Although the participants favoured certain events, who they spent time with was more important than the actual activity.

### Influencing Matters Regarding the Living Environment

All participants wanted to have the authority to influence matters that could contribute to their own or others’ contentment, such as what food to serve at the lunch restaurant, modifications of their apartments or what activities to offer. Those who were confident that they could voice their opinions to staff and management also knew who to turn to and had recent experiences of feeling that their views mattered. But those who felt that staff and management were unavailable said that they were uncertain whether their opinions mattered. One participant commented on this by saying:

There’s one of those suggestion boxes downstairs. I’ve written many letters and have never noticed whether the ideas have been considered (participant 3).

As the above quotation suggests, interaction through letters limited the opportunities for two-way communication because the participant lacked knowledge of if and how the letter was received by the receiver. Having face-to-face interaction instead allowed participants to get immediate feedback on their suggestions, and in most cases their propositions had been implemented, such as cultural exhibitions or what singers to invite to perform at the senior house. Nonetheless, the majority of the participants said that they nowadays lacked the energy to take initiatives to influence, although they knew it was up to them to take the first step. They therefore appreciated when staff and management actively encouraged residential involvement, preferably face-to-face. When the participants talked about what they wished to influence at present, all of them mentioned that they wanted a living environment where residents could do things together and build companionship because it was always the same crowd that attended different activities and the attendance rate at available in-house activities was low, which restricted the opportunities for getting acquainted with other residents.

## Discussion

In this study, we explored what senior housing residents in the age range of 82–97 perceive as social participation that is meaningful and contributes to a meaningful everyday life. Our analysis shows that the six study participants’ perceptions of such involvement encompassed reciprocal social interactions with caring people they connected with and could be themselves around, maintaining control over their social life and having the opportunity to directly communicate with decision makers at the senior house. Overall, the participants’ descriptions of social participation that was meaningful related to various aspects of feeling significant as a person. Together, these aspects formed the overarching theme, *I matter*.

One dimension of feeling significant that our participants talked about related to being caring towards those who needed practical or emotional support or, vice versa, receiving support from people who genuinely cared and wanted to be there for them because the former made them feel appreciated and the latter that they were looked after. Such interactions allowed the participants, as well as those they interacted with, to take on a caring role that was perceived as meaningful. To have the opportunity to take on meaningful roles within the family, community or beyond generates a sense of value and belonging (WHO, 2015). Informal support allowed our participants and their fellow residents, even those in poor health, to maintain social participation they perceived as meaningful. Social participation provides meaning in life and can enable older adults to cope with age-related changes (Palmes et al., 2021).

All participants said that they wanted to strengthen the companionship among the residents, partly because they felt lonely due to the deaths of close friends that used to provide companionship. A common push factor for older adults to relocate to senior housing is to reduce the risk of loneliness (Tyvimaa & Kemp, 2011), but recent research shows that moving to a senior house may increase feelings of loneliness over time (Lotvonen et al., 2018), and senior housing residents in general appear to feel lonelier than their community-dwelling counterparts (Lahti et al., 2021). It is therefore important that the social environment in senior housing encourages residents to interact with one another, as these new social contacts potentially can develop into friendships and thus reduce loneliness (Carroll & Qualls, 2014; Lotvonen et al., 2018; Tyvimaa, 2010).

It was also conveyed by our participants that feeling significant as a person included spending time together with others they felt connected with because they were accepted for who they were and therefore could be themselves, which contributed to a sense of belonging. Nevertheless, in line with previous research, our participants said that they nowadays belonged less because most of their friends had died; consequently, they had a desire to build new social connections (Duppen et al., 2020), particularly with their peers at the senior house. They, however, considered it challenging to create bonds with other residents, partly because there were few opportunities to get acquainted because of the low attendance rate at available in-house activities but also because it was important that they had something in common with those they spent much time with. A variety of organised activity options can facilitate the development of new friendships with people who have similar interests as well as enhance social integration (Roberts & Adams). It is therefore important that available activities encourage a larger percentage of senior housing residents to participate, as such actions can contribute to a more inclusive environment (Fang et al., 2016).

Creating an appealing social environment could, according to our participants, strengthen the sense of companionship and potentially reduce the feelings of loneliness that they all experienced. Although a common push factor to relocating to a senior house is fear of loneliness (Tyvimaa & Kemp, 2011), residents in senior houses appear to feel lonelier than their community-dwelling counterparts (Lahti et al., 2021). Optimising the opportunities for social participation among senior housing residents can reduce feelings of loneliness (Jeste et al., 2019; Jolanki, 2021; Tyvimaa & Kemp, 2011). Yet another reason to take action to prevent loneliness in senior housing residents is the increased occurrence of extreme pandemics such as COVID-19 (Marani et al., 2021), which raises concern for increased loneliness among older adults because of a lack of social participation (Berg-Weger & Morley, 2020). Professions that often come in contact with older adults must take advantage of their experiences from the ongoing pandemic and more systematically use available tools and strategies to prevent loneliness and help older adults to stay connected during future pandemics (Berg-Weger & Morley, 2020).

The most prominent feature of feeling significant that our participants emphasised was that they had the authority to decide the level of social participation, without any interference from others, which strengthened the sense of being in control of their lives (Jolanki, 2021). Having the authority and possibility to make autonomous choices significantly influences older adults’ dignity (WHO, 2015) and the perception of being old (Liu et al., 2020). While it was important to have the opportunity to interact with others, the participants wanted to be in control of when, where and with who they interacted. Making such selective choices allowed them to balance social participation with privacy (Tyvimaa, 2011) and maintain autonomy (Jolanki, 2021; Lotvonen et al., 2018). The participants furthermore said that one motivation for taking part in activities together with others was to maintain physical and cognitive functions as a means to remain autonomous – regular involvement in social activities decreases the likelihood of frailty in old age (Abe et al., 2020). The desire for autonomy can motivate older adults to improve social participation, which subsequently may contribute to higher-quality interactions (Townsend et al., 2021). Another reason the participants stated for making efforts to remain autonomous was because they feared that they would be forced to relocate to a long-term facility if their physical or cognitive health declined too much, meaning that their current independence and social connections could be jeopardised (Carroll & Qualls, 2014). Clearly, it is important to older adults to remain independent for as long as possible. Therefore, if the intention with independent living communities is to foster independence, it is important that staff in senior houses are familiar with the residents and know their needs (Tyvimaa, 2010). A person-centred approach can foster independence as well as social participation, which points to the importance of an active dialogue with senior housing residents about their wishes, needs and functional ability. The information can then be used to help residents to maintain their level of independence and remove barriers for social participation (De Coninck et al., 2021).

The participants expressed that they felt significant when their ideas and opinions were taken seriously. This was particularly evident when they described whether they had the authority to affect matters that directly had an impact on their own or other senior housing residents’ lives. Having the authority to give their opinion on how to make the living environment more satisfying, such as changing the lunch menu or making suggestions for in-house activities, signalled that their voices mattered, which enhances a sense of belonging and inclusion (Fang et al., 2016). It was obvious that the means of communication played a major role in whether the participants felt that their proposals were getting attention. For example, face-to-face interaction with decision makers was considered to be most efficient, whereas writing letters was the least effective. These examples point to the importance of active involvement − engaging senior housing residents in the planning of their living environment spurs interaction with other residents, increases satisfaction with the offered activities (Tyvimaa, 2011), contributes to new social networks and a sense of meaningfulness and gives them a better understanding of the planning process (Henning et al., 2009). However, the participants stated that they nowadays lacked the energy to take the initiative to influence, due to old age, but still wanted to contribute their opinions if the staff or management asked for their input. It is therefore extremely important to continue to engage older adults, regardless of functional ability, as it may remove barriers for social participation (Cachadinha et al., 2011).

As was stated in the introduction, Levasseur et al.’s (2010) taxonomy comprising six levels of social activity was used in this study to gain insight into participation that was meaningful, according to independently living older adults. It helped us to identify that such participation mainly included interactions on levels four and five but also to some extent on level six. On the fourth level, the participants participated in task-oriented activities in collaboration with others to reach a common goal (Duppen et al., 2020), such as planning dinner parties, solving crossword puzzles or attending cultural events. It was nevertheless not the social activities per se that made the interactions meaningful, but rather, the interactions enabled them to maintain their autonomy, remain active and connect with others. Besides, independent living residents’ involvement on the fourth level represents belonging (Shippee, 2012). Social participation on the fourth level contributes to social integration (Levasseur et al., 2010; Roberts & Adams, 2018), social networking (Ashida et al., 2016; Carroll & Qualls, 2014; Levasseur et al., 2010) and enhanced quality of life (Feng et al., 2020; Jenkins et al., 2002).

Involvement in social activities on the fifth level was characterised by being helpful to others, particularly fellow residents who needed assistance due to poor health or feeling lonely. This type of social participation involves social engagement and may delay frailty (Jeste et al., 2019). Even though all of the participants had reduced functioning themselves, they found it meaningful to care for others and therefore motivated to stay socially active (De Coninck et al., 2021). The participants’ attentiveness to other peoples’ needs can be described as altruism and may improve quality of life (Roberts & Adams, 2018). Social engagement promotes personal satisfaction (Aroogh & Shahboulagh, 2020) and a sense of connectedness (Mohler & Miller, 2020) and enables senior housing residents with reduced functional ability to participate socially and maintain a sense of belonging (Roberts & Adams, 2018).

In our study, involvement in social activities perceived as meaningful on the sixth level related to a desire to be actively engaged in issues relating to the senior house because it provided an opportunity to influence the development of their living environment. Even if there were fewer examples of social participation on level six than the previous levels, it was important for us to include these examples to highlight the importance of supporting engagement on this level; it enables older adults to contribute more broadly to society (Duppen et al., 2020) and may benefit many people in the community (Levasseur et al., 2010). The participants’ proposed changes of their local environment could potentially benefit others, both within the senior housing sphere and outside, as some of the services were accessible to the public and certain proposals could be implemented at other senior houses. Just as on the previous level, involvement is characterised by active non-obligatory meaningful social engagement (Levasseur et al., 2010). Broader community social engagement is associated with the sense of living a meaningful life (Amagasa et al., 2017; Steptoe & Fancourt, 2019).

### Methodological Considerations

Several limitations to this study need to be acknowledged. The participants were recruited from one senior housing facility, and this may limit the transferability of the findings to other settings. Less than half of the residents from the senior house we recruited from chose to take part in our study, which resulted in a small study population. A possible reason for the lack of interest is that many of the residents had pronounced decline in functional ability and felt they lacked the energy to participate. Another reason could be that they were less active socially than those who participated. Our participants were older adults in the ‘fourth age’, 80 years and older, and their perceptions of involvement in social activities that is meaningful may be different than younger age groups. Additionally, only one man participated in the present study, which may have resulted in missing viewpoints because previous research has reported gender differences in social participation (Amagasa et al., 2017; De Oliveira et al., 2021; Liu et al., 2020). Thus, a higher percentage of male participants could have generated additional perspectives and resulted in different interpretations of the data.

A key strength of the present study was the thematic analysis process. To achieve a high degree of trustworthiness, a step-by-step approach for conducting a trustworthy thematic analysis, described by Nowell et al. (2017), was employed. Reflexivity was a vital part throughout the research process to ensure ethical compliance and awareness of personal biases (Berger, 2015). Excel spreadsheets were used as a means to document the analytic process and constituted an audit trail of the findings (Nowell et al., 2017). The interpretations and findings were furthermore discussed with our participants during several phases of the analysis to ensure that the findings reflected their viewpoints. In addition, the extracted data from the transcripts were independently reviewed by two of the researchers in the present study.

## Conclusion

This study set out to explore what characterises social participation that is meaningful from the viewpoint of independently living older adults. The findings showed that it encompasses feeling significant as a person – that is, to matter. On a more detailed level, it meant providing reciprocal support to each other, which in turn promoted social participation as well as new friendships. Reciprocal support also facilitated independence, enabling residents to remain in their current home for longer, instead of having to face another move to a long-term care facility and ultimately lose their independence. To promote involvement in social activities perceived as meaningful in senior housing contexts, residents must know that their voices matter and have the authority to influence matters that concern their everyday life or living environment.

Social participation considered meaningful furthermore meant interactions that fostered companionship and reduced feelings of loneliness. Many senior housing residents feel lonely, but a living environment that encourages social participation can prevent loneliness. This is particularly important as extreme pandemics such as COVID-19 will become more frequent in the future. It is thus important to create societies where older adults easily can connect with others and remain connected during periods of social restrictions.

The insights gained from this study may be of assistance to promote older adults’ involvement in social activities that creates a sense connectedness, a sense of belonging and enhanced quality of life; such involvement may contribute to social integration, social networking and involve social engagement. More information on what makes social participation meaningful would help us to establish a greater degree of accuracy on this matter. Further research might explore social participation from a life course perspective, preferably using a longitudinal research design, as it can provide information on how such interactions change over time and their contributing factors. The findings of future research will contribute to a deepened understanding of how social participation can be promoted in old age to make life more meaningful and enhance quality of life.

## Data Availability

Analysed and interpreted data will be anonymized and saved in the Finnish Social Science Data Archive after study completion.
